# Epigenetic Drugs Splitomicin, Suberohydroxamic Acid, CPTH6, BVT-948, and PBIT Moderate Fibro-Fatty Development in Arrhythmogenic Cardiomyopathy

**DOI:** 10.3390/biom15111565

**Published:** 2025-11-06

**Authors:** Melania Lippi, Silvia Moimas, Luca Braga, Yohan Santin, Arianna Galotta, Mauro Giacca, Giulio Pompilio, Elena Sommariva

**Affiliations:** 1 Centro Cardiologico Monzino IRCCS, 20138 Milan, Italy; melania.lippi@cardiologicomonzino.it (M.L.); yohan.santin@cardiologicomonzino.it (Y.S.); arianna.galotta@cardiologicomonzino.it (A.G.); giulio.pompilio@cardiologicomonzino.it (G.P.); 2 Department of Medicine and Surgery, Università degli Studi di Milano Bicocca, 20126 Milan, Italy; 3 International Centre for Genetic Engineering and Biotechnology (ICGEB), 34139 Trieste, Italy; silviamoimas@gmail.com (S.M.); luca.braga@icgeb.org (L.B.); mauro.giacca@kcl.ac.uk (M.G.); 4 British Heart Foundation Centre of Research Excellence, School of Cardiovascular Medicine and Sciences, King’s College London, London WC2R 2LS, UK; 5 Department of Biomedical, Surgical and Dental Sciences, Università degli Studi di Milano, 20122 Milan, Italy

**Keywords:** arrhythmogenic cardiomyopathy, epigenetic drugs, adipogenesis, fibrosis

## Abstract

Arrhythmogenic cardiomyopathy (ACM) is a cardiac disorder manifesting through electrical and contractile dysfunction of the ventricles, characterized by fibro-fatty substitution of the myocardium. Cardiac mesenchymal stromal cells (CMSCs) are key contributors to this remodeling. In clinical management, several pharmacological approaches address ACM arrhythmias and heart failure, but, to date, none specifically target fibro-adipose replacement. Despite genetic origin, several studies have reported that non-genetic aspects influence ACM phenotype, including epigenetic factors. Little is known about their mechanisms in ACM and their potential therapeutic applications. In this work, we aimed to test whether, by perturbing the epigenetic landscape of ACM CMSCs, we could influence their propensity to fibro-fatty differentiation. We conducted a hypothesis-free screening of 157 epigenetic drugs on CMSCs, isolated from ACM patients. Through fluorescence assays, we evaluated lipid droplet accumulation, collagen deposition, and cell viability. Of the 157 drugs screened, five (splitomicin, suberohydroxamic acid, CPTH6, BVT-948, and PBIT) attenuated adipogenic differentiation of ACM CMSCs, with BVT-948 and CPTH6 also reducing collagen production. Overall, this study identified specific epigenetic drugs that were effective in reducing the fibro-fatty phenotype of ACM stromal cells, thus offering potential for adjunctive therapies in the clinical management of ACM patients.

## 1. Introduction

Arrhythmogenic cardiomyopathy (ACM) is a rare genetic cardiac disorder with an approximate prevalence of one in five thousand individuals [1]. ACM predominantly affects the right ventricle leading to structural, morphological, functional, and electrical abnormalities. Imaging studies reveal ventricular dilation, systolic dysfunction, and wall motion abnormalities. Patients can range from exhibiting mild electrocardiographic (ECG) alterations to experiencing life-threatening ventricular arrhythmias, such as ventricular tachycardia, fibrillation, or sudden cardiac death. The distinguishing hallmark of ACM is the atrophy of the ventricular myocardium, which is progressively replaced by fatty tissue and fibrosis [2]. ACM cardiac mesenchymal stromal cells (CMSCs) are the primary source of fibro-adipose substitution, undergoing pathological differentiation into adipocytes and myofibroblasts in the hearts of affected patients [3,4]. This tissue remodeling exacerbates both contractile and electrical dysfunctions, potentially leading to heart failure and arrhythmias [5].

Current pharmacological strategies for ACM focus on symptom management, with beta-blockers and Class III antiarrhythmics to control arrhythmias, while sartans or ACE inhibitors are mainly used for heart failure management [6]. To date, the available treatments have not been specifically designed to attenuate or prevent fibro-adipose substitution, which plays a key role in pathological tissue remodeling.

The genetics of ACM is mainly characterized by autosomal dominant inheritance and presence of pathogenic variants in desmosomal genes (i.e., *PKP2*, *DSP*, *JUP*, *DSG2*, *DSC2*), which are the most frequently mutated in these patients [7]. However, the causative genetic defects are identified in only ~50% of cases, including both familial and non-familial forms [8].

The penetrance and expressivity of ACM are highly variable and, to date, different genetic and non-genetic factors influencing the manifestation of the disease have been described as useful tools for risk stratification. Beyond the main effect of causative mutation(s) on the phenotype [9,10,11], some genetic polymorphisms can modulate specific clinical parameters [12].

Among non-genetic contributors, different players influence the prognosis of the pathology, including hormone levels [13], intense physical exercise [14], and lipid oxidation [15]. Furthermore, a recent study conducted in our laboratory suggested that epigenetic machinery may contribute to the pathogenesis of ACM, indicating a potential target for intervention [16].

Epigenetics consists of chemical alterations in DNA and histones that modulate gene expression without changes in DNA sequence. Epigenetic regulation is mainly based on the availability of promoter regions for binding by transcription factors. Chromatin can switch from a condensed state that restricts gene expression, to a relaxed state in which gene transcription can proceed. The fundamental unit of chromatin is the nucleosome, composed of an octamer of histone proteins, which, through specific modifications, allows chromatin remodeling. The intricate interaction among various epigenetic regulators, including histone deacetylases (HDACs), histone acetyltransferases (HATs), lysine methyltransferases (KMTs), lysine demethylases (KDMs), and DNA methyltransferases, regulates methylation of DNA and acetylation and methylation of histones within nucleosomes. These functions are crucial for organizing chromatin architecture and shaping the transcriptional landscape, often responding to environmental influences. Generally, DNA methylation represents a key mechanism for gene silencing, mainly at promoters and transposable elements. Conversely, demethylation and deacetylation of histones lead to chromatin condensation and result in repression of gene expression [17]. In addition to these epigenetic enzymes, non-coding RNAs, including microRNAs, long non-coding RNAs, small interfering RNAs, and circular RNAs, play a modulatory role within epigenetic machinery, contributing to the regulation of gene expression [17,18,19].

While epigenetics underlies processes central to ACM, such as cardiac fibrosis [20], adipogenesis [21], and arrhythmia onset [22], research on epigenetic mechanisms/therapies in ACM is still scarce. In contrast, epigenetic drugs (epidrugs) are being tested for other cardiovascular diseases, both preclinically and in clinical trials [23,24,25].

In this study, we tested whether perturbing the epigenetic landscape could modulate the fibro-fatty remodeling that characterizes ACM. Using patient-derived CMSCs, which are an *in vitro* model that faithfully recapitulates ACM phenotypes [3,4], we performed a hypothesis-free screening of 157 epigenetic drugs. This approach uncovered a subset of compounds capable of attenuating adipogenesis and collagen deposition, highlighting epigenetic modulation as a promising therapeutic avenue for ACM.

## 2. Materials and Methods

### 2.1. ACM Population

This study was conducted in accordance with the declaration of Helsinki and was approved by the Ethics Committee of Istituto Europeo di Oncologia and Centro Cardiologico Monzino on 3 July 2019 (R1020/19-CCM1072). The studied ACM population included five unrelated patients recruited at Centro Cardiologico Monzino IRCCS from 2014 to 2023. All patients met the 2010 International Task Force Criteria. Each ACM patient provided written informed consent for the donation of right ventricular endomyocardial biopsy samples. Patient characteristics are summarized in Table S1. The cohort is composed by three males and two females, with an average age of 39 ± 6 years old. The clinical manifestation was heterogeneous, but all patients displayed fibro-adipose tissue remodeling as detected by cardiac magnetic resonance or histological characterization of endocardial biopsy. All subjects experienced arrhythmic episodes, but with variable severity, spanning from sporadic premature ventricular contractions to arrhythmic storm. Four out of five patients present kinetic abnormalities and ventricular dilation and/or systolic dysfunction, whereas one carried a pathogenic mutation in an ACM-associated gene.

### 2.2. CMSC Isolation and Maintenance

CMSCs were obtained by digestion of endocardial biopsies, and the mesenchymal phenotype was confirmed by immuno-phenotypical characterization, as previously described [26]. CMSCs were cultured in maintenance medium (MM), consisting of Iscove’s Modified Dulbecco’s Medium (IMDM, Thermo Fisher Scientific, Waltham, MA, USA) supplemented with 20% of fetal bovine serum (FBS; EuroClone, Pero, Italy), 10 ng/mL of basic fibroblast growth factor (Peprotech, London, UK), 10,000 U/mL of penicillin (EuroClone), 10,000 μg/mL of streptomycin (EuroClone), and 0.02 M of L-glutamine (EuroClone). Cells between passages 3 and 6 were used for all experiments.

### 2.3. CMSC Differentiations

For differentiations, CMSCs were detached with trypsin-EDTA (EuroClone). After trypsin inactivation with FBS, cells were collected and centrifuged at 400× *g* for 5 min at room temperature. The pellet was resuspended in MM and cells were seeded into 96-multiwell plates (PerkinElmer, Waltham, MA, USA) optimized for fluorescence detection by Operetta^®^ (PerkinElmer) at a final density of 4.7 × 10^4^ cells/cm^2^. After 24 h of cell attachment, CMSCs were cultured for 6 days in either adipogenic [3] or pro-fibrotic [4] media, as previously described, with media refreshed every 72 h. For adipogenic stimulation, cells were cultured in IMDM supplemented with 10% of FBS, 0.1 of mM indomethacin (Sigma Aldrich, St. Louis, MO, USA), 1 μM of hydrocortisone (Sigma Aldrich), and 0.5 mM of 3-isobutyl-methylxanthine (Sigma Aldrich). For myofibroblast activation, cells were first starved for 5 h in MM containing only 2% FBS, followed by the addition of TGFβ1 5 ng/mL (Peprotech) to the same medium. Throughout the six-day differentiation period, cells were exposed to the drugs as detailed in the following section.

### 2.4. Epigenetic Drug Treatments

The epigenetic drug library (Cayman) consisted of 157 epigenetic compounds dissolved in DMSO, distributed in two 96-well plates, optimized for high-throughput screening. This library was selected for its comprehensive and balanced composition of small molecules targeting a wide range of epigenetic regulators, enabling systematic exploration of epigenetic modulation mechanisms. CMSCs were treated with epidrugs at the appropriate concentrations, added to the differentiation media at the start of differentiation and again at 72 h during media refresh, for a total of 6 days. The list of the epigenetic drugs, and the experimental setup are detailed in the Supplementary Materials section (Table S2). For the screening, CMSCs from two ACM patients (ACM1 and ACM2) were treated with each drug at 1 μM. 1% of DMSO-treated cells were used as reference controls. The hit validation was performed by treating CMSCs derived from five ACM patients with the selected drugs at multiple concentrations, in technical duplicate. Doses were chosen based on the literature [27,28,29,30,31,32,33,34,35,36].

### 2.5. Fluorescence Assays

Nuclei count, lipid droplet accumulation, and collagen deposition were evaluated through fluorescence assays. After differentiation and treatment, CMSCs were fixed with 4% of paraformaldehyde (Sigma Aldrich) in phosphate-buffered solution (PBS, EuroClone) for 10 min. Blocking and permeabilization were performed in PBS supplemented with 3% of bovine serum albumin (BSA, Sigma Aldrich) and 0.1% of Triton X-100 (Sigma Aldrich) for 60 min. For collagen staining, cells were incubated overnight at 4 °C with primary antibody anti-COL1A1 (Cell Signaling Technology) diluted 1:200 in PBS with 3% of BSA (PBS/BSA), followed by a secondary antibody (1:200 dilution), and 10 µg/mL of Hoechst 33342 (Sigma-Aldrich) for nuclei staining. For lipid staining, cells were labeled with 0.5 µg/mL of Nile Red (Sigma Aldrich) and 10 µg/mL of Hoechst in PBS/BSA. After incubation of 60 min in the dark at room temperature for both conditions, images were acquired with the appropriate filters using the Operetta^®^ fluorescence microscope and analyzed using Harmony software 5.1 (PerkinElmer).

### 2.6. Statistical Analysis

Quantitative results are expressed as mean ± standard error of the mean (SEM). Comparisons between treated and control groups were performed by paired two-tailed *t*-test using GraphPad Prism 10. Differences were considered statistically significant at *p* value ≤0.05.

## 3. Results

### 3.1. Epigenetic Drug Screening

**Figure 1:**
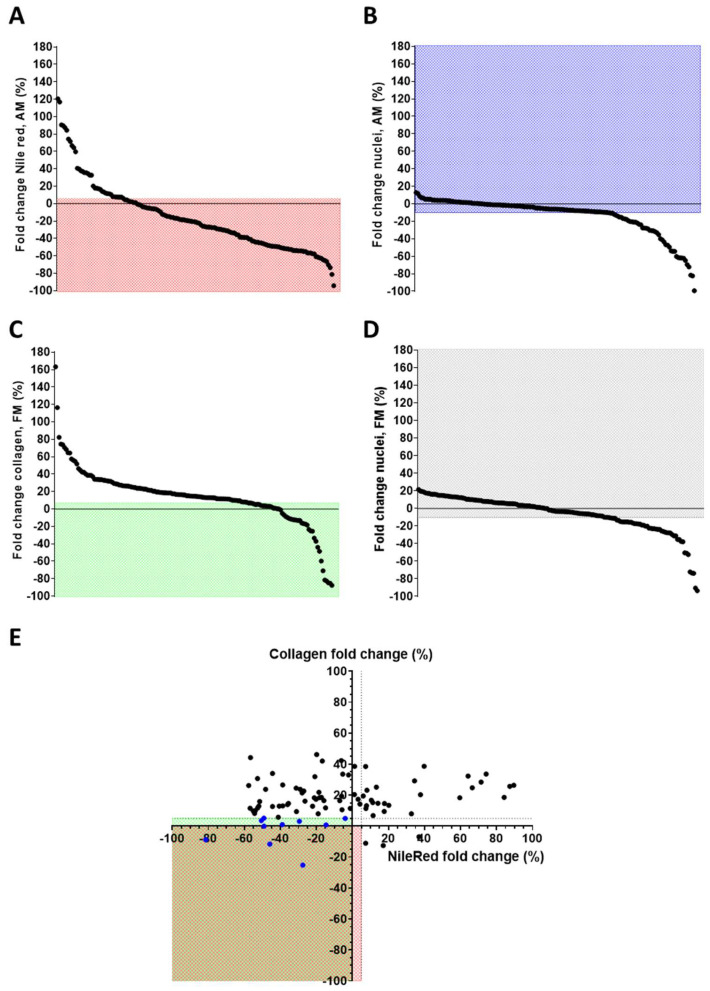
Epigenetic drug screening. Graphical representation of the effect of epigenetic drug screening on ACM CMSCs. (**A**) Fold change in Nile Red signal quantification of CMSCs in adipogenic medium (AM) treated with epidrugs, using the mean of DMSO-treated as reference; in the red field are the drugs inhibiting lipid accumulation or not increasing it (≤+5% compared to controls). (**B**) Fold change in nuclei number of CMSCs under adipogenic stimuli treated with epidrugs; in the blue field are the non-toxic drugs (nuclei count > −10% compared to controls). (**C**) Fold change in collagen signal quantification of CMSCs in pro-fibrotic medium (FM) with epidrugs, using the mean of DMSO-treated as reference; in the green field are the drugs inhibiting collagen accumulation or not increasing it (≤+5% compared to controls). (**D**) Fold change in nuclei number of CMSCs under pro-fibrotic stimuli with epidrugs; in the gray field are the non-toxic drugs (nuclei count > −10% compared to controls). The mean of DMSO-treated controls was used as reference. (**A**–**D**) The spots represent the screened compounds sequentially, ordered in decreasing order of the y-axis values. (**E**) Combined fold changes in Nile Red and collagen signals (in the red or green field, respectively) of non-toxic epigenetic drugs; blue spots represent the drugs able to inhibit the adipose or fibrose phenotype, without enhancing the other one over 5% (**E**). Means of biological replicates are shown (*n* = 2 for each drug). Nile red and collagen signals were normalized on nuclei number.

For the drug screening phase, CMSCs derived from two patients, ACM1 and ACM2 (see Table S1 for patients’ characteristics), were treated with 157 compounds from the library (Table S2) under either adipogenic or pro-fibrotic stimulation. Cell viability (assessed via nuclei count) and markers of fibro-adipose differentiation were evaluated. Figure 1 shows the fold change relative to DMSO-treated controls for lipid accumulation and collagen deposition with the corresponding nuclei counts. In the adipogenic medium, among the 157 compounds screened, 119 showed a decrease in lipid droplet amount (Figure 1A). Of the total, 50 drugs were excluded due to cytotoxicity (Figure 1B). Ultimately, 74 non-cytotoxic epidrugs demonstrated the ability to inhibit adipogenic differentiation or did not promote it beyond a tolerance of 5%. Under pro-fibrotic stimulation, 38 of 157 drugs were able to reduce collagen deposition (Figure 1C). However, 52 compounds were excluded due to cytotoxicity (Figure 1D). As a result, 20 non-cytotoxic compounds were effective in reducing fibrosis, or did not increase it beyond a 5% tolerance.

To identify promising therapeutic candidates, we prioritized non-toxic drugs that reduced one of the two phenotypes without concomitantly increasing the other by more than +5% (Figure 1E). Ten drugs fulfilled this criterion: splitomicin, SBHA, CPTH6, GSK126, EPZ5676, BVT-948, NI-57, PBIT, CAY10722, and CPTH2 (Table 1). A summary of the screening outcome is provided in Table S3, while the flowchart of the screening results is represented in Figure S1.

### 3.2. Validation of Screening Hits

**Figure 2:**
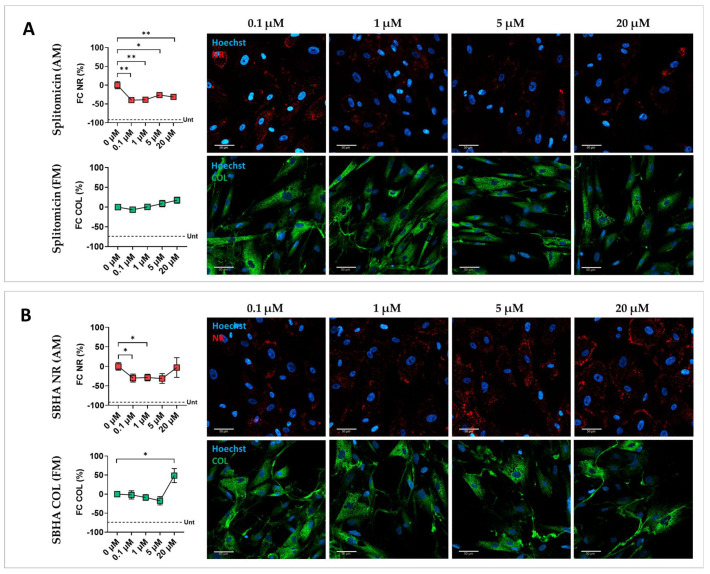

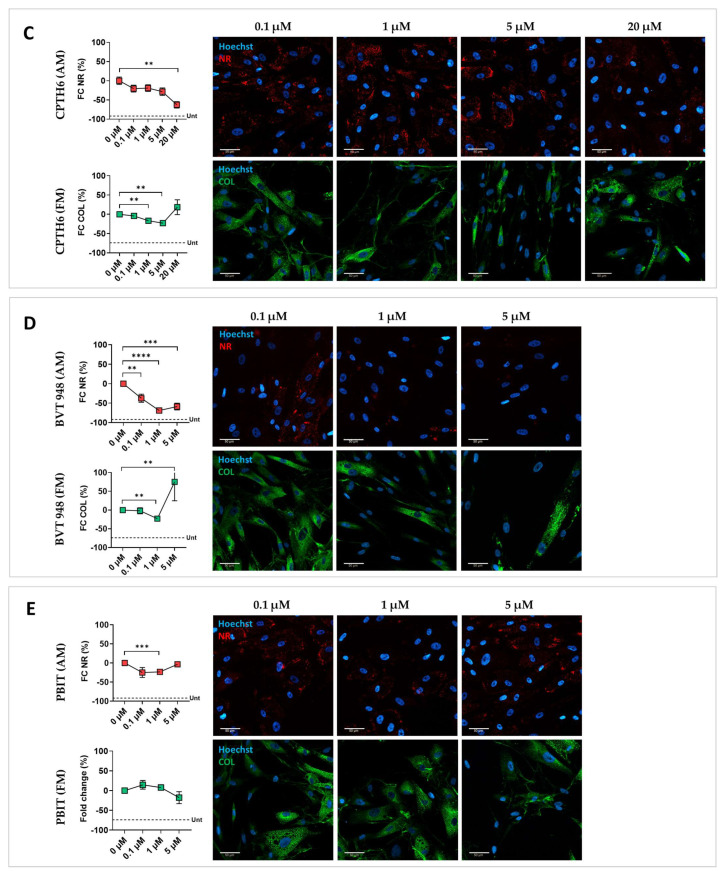
Effect of splitomicin, SBHA, CPTH6, BVT-948, and PBIT on ACM CMSCs. Graphical representation of the effect of the effective epigenetic drugs on ACM CMSCs from five patients (ACM1-5), with the relative representative fluorescent images. (**A**) Fold change (FC, %) in Nile Red (NR, in red) or collagen (COL, in green) signal quantification normalized on nuclei number and representative fluorescent images for CMSCs treated with splitomicin; (**B**) suberohydroxic acid (SBHA); (**C**) CPTH6; (**D**) BVT-948; (**E**) PBIT. The mean of DMSO-treated controls in AM and FM was used as reference and set to zero; the mean of the DMSO-treated controls in MM is represented by a dotted line (Unt, untreated in **A**–**E**). In the graphs, each dot represents the mean of biological replicates ± standard errors (*n* = 5). A paired two-tailed *t*-test was used for comparisons: * = *p* value <0.05; ** = *p* value < 0.005; *** = *p* value < 0.0005; **** = *p* value < 0.0001. Magnification: 40×; scale bar: 50 µm.

To confirm screening results and account for inter-individual variability, a validation step was performed on CMSCs derived from five unrelated ACM patients (Table S1). Each drug was tested at different concentrations to identify the optimal dose capable of moderating the fibro-fatty phenotype without toxic effects. A dose–response analysis of the 10 selected compounds was carried out, with concentrations chosen based on the literature data [27,28,29,30,31,32,33,34,35,36]. Among the 10 tested drugs, only five were confirmed to be effective across all five patients. Graphical representations of the signal quantification and representative fluorescence images illustrating lipid and collagen staining across treatment conditions of the effective drugs are shown in Figure 2. CMSCs treated with DMSO in MM were used as undifferentiated controls (see Figure 2A and Figure S2). CMSCs treated with DMSO in AM and FM were used as reference controls for adipogenic and fibrotic readouts, respectively (Figure 2B). As shown in Figure 2C, splitomicin consistently attenuated lipid accumulation at all tested doses, compared with controls, with a reduction in cell viability only at 20 µM (Figure S3), and without any notable effect under pro-fibrotic conditions. Suberohydroxamic acid (SBHA) significantly inhibited lipid accumulation at lower doses, but at higher concentrations caused cytotoxicity and an increase in collagen content (Figure 2D and Figure S3). CPTH6 treatment effectively inhibited both adipogenesis and collagen production, accompanied by a slight increase in cell number (Figure 2E and Figure S3). BVT-948 inhibited lipid accumulation at all tested concentrations and significantly reduced collagen deposition at 1 µM (Figure 2D). However, at 5 µM, the drug showed a pronounced toxic effect (Figure S3). PBIT treatment successfully attenuated adipogenic differentiation at 1 µM, without affecting fibrosis (Figure 2E). These findings are summarized in Table 2, which provides a comparative overview of phenotypic outcomes and toxicity profiles for each validated compound.

Among the ten hits, five did not confirm the screening results. Neither GSK126 nor EPZ5676 displayed significant modulation of either phenotype. The beneficial effects of NI-57 and CAY10722 were not verified, and cell death occurred at high doses. CPTH2, decreased collagen at 5 µM but also markedly increased lipid content, disqualifying it as a viable candidate. These findings are summarized in Figure S4 and Table S4.

## 4. Discussion

This work shows for the first time that targeted epigenetic modulation can blunt the fibro-fatty remodeling that characterizes arrhythmogenic cardiomyopathy (ACM). From a screen of 157 compounds in patient-derived cardiac mesenchymal stromal cells (CMSCs)—a model that recapitulates adipogenic and pro-fibrotic outputs in ACM [3,4]—we identified five agents (splitomicin, SBHA, CPTH6, BVT-948, and PBIT) that consistently mitigated one or both disease-relevant phenotypes. These observations align with prior evidence that epigenetic remodeling contributes to ACM pathology and stromal cell-driven remodeling [16,37,38].

Interestingly, our data highlight that deacetylase inhibition is an effective lever to prevent adipogenesis. A clear example is splitomicin, which is an inhibitor of class III histone deacetylases (HDACs), also known as sirtuins. Splitomicin reproducibly reduced lipid accumulation across donors with preserved viability but had no impact on collagen. This effect is consistent with previous reports and fits a model in which sirtuins, through deacetylation of H3K9ac, H3K56ac, and H4K16ac, as well as regulation of non-histone substrates such as PGC-1α, more strongly modulate PPARγ-driven adipogenic programs than pro-fibrotic ones in ACM CMSCs [39,40]. Given the strong adipogenic propensity of these cells, inhibition of sirtuin activity likely dampens pathological PPARγ signaling and limits lipid accumulation, though the lack of anti-fibrotic benefit underscores the complexity and isoform-specific functions of sirtuins [39,41,42]. We next examined the role of zinc-dependent HDACs (classes I, II, and IV) in ACM CMSCs. In this context, SBHA, a hydroxamic acid-based HDAC inhibitor [43], attenuated differentiation of CMSCs toward a fat cell fate, but not activation into myofibroblasts. At higher doses, however, it impaired viability and unexpectedly increased collagen deposition. This supports previous work with pan- and class I/II HDAC inhibitors such as trichostatin A, which block early adipogenesis yet show variable anti-fibrotic outcomes depending on tissue, dose, and context [44,45,46]. Inhibition of multiple zinc-dependent HDAC isoforms has also shown beneficial effects in ACM and other disease contexts. For instance, it was recently shown that Givinostat, a class I and II HDAC inhibitor, can inhibit the fibro-adipose differentiation *in vitro* of fibroadipogenic precursors from an ACM mouse model [47]. In addition, Givinostat is currently under investigation in clinical trials for Duchenne muscular dystrophy (DMD), where it mitigates adipogenesis, inflammation, and fibrosis in murine skeletal muscle [24,25,48,49]. Given the contribution of inflammation to ACM pathogenesis [50], the reported anti-inflammatory effects of SBHA, through reduction in pro-inflammatory cytokines, such as IL6 and TNFα [51,52,53], may also be relevant. These mixed effects highlight the limitations of non-selective inhibition, as individual HDAC isoforms can exert opposing influences on stromal fate decisions. In ACM, this complexity is particularly relevant given the intertwined nature of adipogenic and fibrotic remodeling.

Interestingly, inhibition of CPTH6, an acetylation writer, produced a broader benefit. CPTH6 inhibits the lysine acetyltransferase activity of general control non-repressed 5 protein (GCN5) and p300/CBP-associated factor (pCAF) [54], which are two paralogs. CPTH6 reduced both lipid accumulation and collagen deposition while preserving cell viability. Mechanistically, GCN5/pCAF catalyze H3K9ac and acetylate non-histone substrates, thereby promoting chromatin accessibility at metabolic and TGFβ-responsive loci [55,56,57]. Our findings align with prior ACM evidence of elevated GCN5 activity and reduced lipid accumulation upon its inhibition [58], as well as genetic data showing that dual GCN5/pCAF loss downregulates PPARγ programs in brown preadipocytes [59]. On the fibrotic side, the reduction in collagen is consistent with the broader principle that HAT/HDAC balance governs myofibroblast activation, exemplified by CBP/p300-driven acetylation enhancing SM22α expression in response to TGFβ1 [60].

Our data argue that adipogenesis in ACM CMSCs is highly acetylation-sensitive, whereas fibrosis may depend on distinct acetylation marks or writer-specific mechanisms. Although HATs and HDACs catalyze opposing reactions, the convergence we observe can be explained by the behavior of adipogenic super-enhancers, which operate within a narrow acetylation window [61,62,63]. In this framework, adequate H3K27ac/H3K9ac levels sustain adipocyte gene expression, but shifting acetylation above this setpoint with splitomicin or SBHA, or below it with CPTH6, may destabilize the same regulatory hubs and suppress adipogenesis.

Beyond acetylation, lysine–methyl pathways provided complementary, more selective control of the phenotype. For instance, PBIT, an inhibitor of JARID1/KDM5 histone demethylases [64], effectively reduced adipogenesis but showed only a mild trend toward decreasing fibrosis. *In vitro* studies have reported that KDM5C suppression impairs adipocyte maturation [65], while KDM5B inhibition reduces cardiac fibrosis both *in vitro* and *in vivo* during fibroblast differentiation [66]. In humans and mice, KDM5C expression correlates with body mass index and fat mass, respectively [67]. Our findings are in line with these observations, supporting JARID1/KDM5 inhibition as a promising anti-adipogenic approach. An important nuance concerns BVT-948. At 1 μM, it reduced both lipid accumulation and collagen, whereas higher concentrations eroded anti-fibrotic benefit and introduced toxicity. Although BVT-948 is primarily described as a protein tyrosine phosphatase (PTP)/cytochrome P450 (CYP) inhibitor [68], it has also been reported to inhibit the histone lysine methyltransferase SET8 (KMT5A/PR-Set7) [69]. SET8 is the sole enzyme that monomethylates H4K20 *in vivo*, which is a mark linked to chromatin structure and gene regulation [70,71]. Selective SET8 inhibition has shown anti-fibrotic activity by suppressing myofibroblast markers and promoting dedifferentiation of patient-derived lung myofibroblasts [72]. In this context, our low-dose BVT-948 data are consistent with partial SET8 engagement as a chromatin-level mechanism, whereas the loss of anti-fibrotic signal and toxicity at 5 μM likely reflects broader target engagement. A plausible explanation for these high-dose effects is BVT-948 activity on PTP/CYP. Different PTPs can drive opposite outcomes in adipogenesis and fibrosis. While PTP-BL downregulation represses adipogenesis [73], PTP-RQ overexpression limits adipocyte differentiation [74]. In addition, the loss of PTP1B [75] or low molecular weight PTP (LMW-PTP) [76] reduces post-injury fibrosis, so broader PTP engagement at a higher dose could dilute the anti-fibrotic signal. CYPs, in turn, shape lipid handling through metabolites that modulate nuclear receptors, such as PPAR/LXR [77,78]. Silencing CYP2E1 or CYP2F2 lowers *PPARγ*/*CD36* expression [79,80], while CYP2J2 can be anti-fibrotic [81] and other CYPs become pro-fibrotic under mechanical stress [82]. Taken together, dose-dependent PTP/CYP engagement offers a plausible mechanism for the dual benefit at 1 µM and its reversal at 5 µM.

Collectively, our findings show that epigenetic modulation is a promising pharmacologic approach to control fibro-fatty remodeling in human ACM stromal cells. This is in agreement with our previous work, which indicated an unstable chromatin remodeling in ACM-CMSCs [16]. It is likely that the maintenance of the pathological phenotype in ACM cells is sustained by a hyperdynamic epigenetic state that may constitute a therapeutic opportunity: indeed, by stabilizing a single key node, the balance could be shifted and adipogenesis and/or fibrosis could be suppressed. Thus, epigenetic drugs do not need to normalize the entire epigenome; instead, their therapeutic potential may lie in exploiting specific flaws, as in cancer therapy, where the epigenetically unstable cells can be targeted with selective inhibitors [83]. Nevertheless, epigenetics is intrinsically broad, pleiotropic, and devoid of tissue specificity, and, in a future scenario, careful preclinical and clinical testing will be needed to assess safety, efficacy, off-target effects, and ACM-specificity.

In conclusion, adjusting the acetylation state with selective HAT and HDAC inhibitors consistently suppresses adipogenesis and when biased toward reduced writer activity, it also lowers collagen. Targeting specific methylation targets such as KDM5 or SET8 provides additional leverage on lineage programs. Together, these results provide a strong rationale for developing regimens that combine acetylation and methylation modulators to constrain both adipogenic and pro-fibrotic programs in ACM.

## 5. Conclusions

Our findings highlight the potential of epigenetic modulation as a novel and specific approach to counteract the pathological fibro-fatty remodeling characteristic of ACM. Five compounds (splitomicin, SBHA, CPTH6, BVT-948, and PBIT) were able to selectively attenuate either adipogenic or fibrotic responses in ACM-derived CMSCs, without promoting the other phenotype, and without cytotoxicity, suggesting the existence of distinct and druggable epigenetic pathways underlying the disease. These results provide the first experimental validation that the fibro-fatty substitution in ACM is not only epigenetically regulated but also amenable to pharmacological intervention. While this study represents an initial step, further investigations are warranted to dissect the molecular mechanisms involved and to advance the most promising candidates toward preclinical and clinical development, with the long-term goal of delivering targeted therapies capable of modifying disease progression in ACM patients.

## Figures and Tables

**Table 1:** Selected drugs from the epigenetic drug screening. Table summarizing the effect of the selected epigenetic drugs on ACM CMSCs. Fold change (FC), with respect to DMSO-treated cells, in Nile Red signal quantification, normalized on nuclei number, of CMSCs in adipogenic medium (AM) and in pro-fibrotic medium (FM) treated with epidrugs is expressed as mean of the biological replicates (ACM1 and ACM2). FC, with respect to DMSO-treated cells, in nuclei number of CMSCs in AM and in FM with epidrugs expressed as mean of biological replicates. The mean of DMSO-treated controls was used as reference value.

Epigenetic Drugs	Mean of Fold Change (%) NR/Nuclei Number (AM)	Mean of Fold Change (%) Nuclei Number (AM)	Mean of Fold Change (%) COL/Nuclei Number (FM)	Mean of Fold Change (%) Nuclei Number (FM)
Splitomicin	−4.00	−1.59	5.00	13.18
CPTH2 (hydrochloride)	−27.52	0.36	−25.18	14.11
Suberohydroxamic acid	−14.65	−8.04	0.78	5.52
CPTH6 (hydrobromide)	−50.56	12.87	3.53	19.76
GSK126	−38.94	−5.59	1.02	12.37
EPZ5676	−49.14	3.94	5.00	17.27
BVT 948	−81.41	8.69	−8.70	19.32
NI-57	−49.06	−2.47	−0.02	21.58
PBIT	−29.46	−9.30	3.11	14.21
CAY10722	−45.88	1.55	−11.63	−4.14

**Table 2:** Effect of splitomicin, SBHA, CPTH6, BVT-948, and PBIT on ACM CMSCs. The table summarizes the effect of the successful epigenetic drugs on ACM CMSCs from five patients (ACM1-5). Fold change (FC) percentage in Nile Red signal quantification normalized on nuclei number of CMSCs in adipogenic medium (AM) and of collagen (COL) in pro-fibrotic medium (FM) treated with epidrugs is expressed as mean of biological replicates ± standard error; the mean of the controls in AM and FM was used as reference value. Fold change percentage in nuclei number of CMSCs in AM and in FM with selected epidrugs is expressed as mean of biological replicates; the mean of DMSO-treated controls in AM and FM was used as reference value (0%).

Epigenetic Drug	Dose	FC (%) Nile Red/Nuclei Number (AM)		FC (%) Nuclei Number (AM)		FC (%) COL/Nuclei Number (FM)		FC (%) Nuclei Number (FM)	
		**Mean**	**SEM**	**Mean**	**SEM**	**Mean**	**SEM**	**Mean**	**SEM**
Splitomicin	0.1 μM	−39.8	5.7	−2.2	1.5	−6.6	6.3	−1.4	2.0
	1 μM	−38.8	6.8	−2.2	1.6	0.6	5.5	0.3	0.7
	5 μM	−26.2	4.8	−2.9	1.6	8.9	8.0	1.8	2.6
	20 μM	−31.2	3.5	−11.3	3.8	17.5	7.7	−3.4	2.9
SBHA	0.1 μM	−29.9	10.2	2.1	1.0	−2.0	10.7	3.7	1.6
	1 μM	−28.6	8.5	1.0	1.5	−8.8	7.5	3.5	2.2
	5 μM	−31.1	12.6	−5.1	0.9	−17.4	11.0	−0.1	3.0
	20 μM	−3.0	25.2	−16.7	1.8	48.8	18.5	−15.7	4.4
CPTH6	0.1 μM	−20.5	−19.1	0.6	1.1	−4.3	5.1	4.5	2.2
	1 μM	8.8	8.6	6.7	2.5	−16.8	3.2	13.6	1.5
	5 μM	−20.5	−19.1	1.9	1.1	−22.9	3.9	13.8	2.8
	20 μM	8.8	8.6	−8.4	2.6	18.3	18.3	−5.5	6.8
BVT 948	0.1 μM	−37.3	10.3	−0.8	2.6	−1.9	8.2	12.4	3.4
	1 μM	−69.0	6.3	0.6	2.1	−23.0	6.1	14.6	8.1
	5 μM	−58.8	8.7	−11.5	3.1	75.3	50.5	−46.7	12.3
PBIT	0.1 μM	−25.0	12.2	−2.4	2.0	14.6	10.9	−5.6	1.8
	1 μM	−23.3	3.8	−0.9	1.4	7.8	6.9	−3.3	2.6
	5 μM	−3.4	6.1	−5.9	2.8	−17.8	15.1	−17.5	10.1

## Data Availability

The original contributions presented in this study are included in the article/Supplementary Materials. Further inquiries can be directed to the corresponding author.

## Supplementary Materials

The following supporting information can be downloaded at https://www.mdpi.com/article/10.3390/biom15111565/s1, Table S1: ACM population; Table S2: Epigenetic drug library; Table S3: Epigenetic drug screening; Table S4: Effect of GSK126, EPZ5676, NI-57, CAY10722, and CPTH2 on ACM CMSCs; Figure S1: Drug screening flow chart; Figure S2: Nile red and collagen signal of DMSO-treated CMSCs in maintenance medium and under adipogenic or pro-fibrotic stimuli; Figure S3: Effect of splitomicin, SBHA, CPTH6, BVT-948, and PBIT on nuclei count of ACM CMSCs; Figure S4: Effect of GSK126, EPZ5676, NI-57, CAY10722, and CPTH2 on ACM CMSCs.

## Abbreviations

The following abbreviations are used in this manuscript:

ACM	Arrhythmogenic cardiomyopathy
BSA	Bovine serum albumin
BVT-948	4-hydroxy-3,3-dimethyl-2H-benz[g]indole-2,5(3H)-dione
CAY10722	N-[2-(2,4-dichlorophenyl)-5-benzoxazolyl]-benzeneacetamide
CMSC	Cardiac mesenchymal stromal cells
CPTH2	(E)-4-(4-chlorophenyl)-2-(2-(3-methylcyclopentylidene)hydrazinyl)thiazole hydrochloride
CPTH6	(E)-4-(4-chlorophenyl)-2-(2-(3-methylcyclopentylidene)hydrazinyl)thiazole hydrobromide
CYP	Cytochrome P450
DMD	Duchenne muscular dystrophy
ECG	Electrocardiography
EPZ-5676	(Pinometostat) (2R)-2-[(1R)-1-[5-[(4-cyclopropylpiperazin-1-yl)methyl]-1H-indazol-3-yl]-2-methylpropyl]-N,6-dimethyl-1,2-dihydro-1,3,5-triazine-4-carboxamide
epidrugs	Epigenetic drugs
FBS	Fetal bovine serum
GCN5	General control non-repressed 5 protein
GSK126	4-(4-methylpiperazin-1-yl)-N-[1-(1-methylpyrazol-4-yl)cyclopentyl]-2-[(1H-pyrrolo [2,3-b]pyridin-3-yl)methyl]benzamide
HAT	Histone acetyltransferase
HDAC	Histone deacetylases
KDM	Lysine demethylase
KMT	Lysine methyltransferase
MM	Maintenance medium
NI-57	(R)-3-(2-(1H-indol-3-yl)ethyl)-N-(4-(pyrrolidin-1-ylmethyl)phenyl)-1H-pyrrolo[2,3-b]pyridine-5-carboxamide
PBIT	2-4(4-methylphenyl)-1,2-benzisothiazol-3(2H)-one
PPAR	Peroxisome proliferator-activated receptor
pCAF	p300/CBP-associated factor
PTP	Protein tyrosine phosphatases
SBHA	Suberohydroxamic acid
