# Supplementary material for: Epigenetic Drugs Splitomicin, Suberohydroxamic Acid, CPTH6, BVT-948, and PBIT Moderate Fibro-Fatty Development in Arrhythmogenic Cardiomyopathy
Source: Biomolecules. 2025 Nov 6;15(11):1565. doi: 10.3390/biom15111565 (PMC12650369; doi:10.3390/biom15111565)
Supplement: Supplementary file 1 [file biomolecules-15-01565-s001.zip › biomolecules-3939114-supplementary.pdf]

Supplementary Materials

Table S1. ACM population. Summary of clinical characterization of the involved patients affected by arrhythmogenic cardiomyopathy. RV: right ventricle; ECG: electrocardiography; n.a.: not available; LBBB: left bundle branch block; VT: ventricular tachycardia; PVC: premature ventricular contractions.

Patient code	Sex	Age	RV dysfunction and structural alterations	Tissue characterization of RV wall	ECG abnormalities	Arrhythmias	Family history (ACM, SCD)
**ACM1**	female	18	dyskinesia, dilation and functional dysfunction	fibro-adipose substitution	inverted T waves in V1,V2,V3 leads	LBBB morphology VT	negative family history, no genetic mutations
**ACM2**	male	38	dyskinesia and dilation	fibro-adipose substitution	inverted T waves in V1,V2 leads, TAD in V2	LBBB morphology VT	negative family history, no genetic mutations
**ACM3**	male	57	dyskinesia and functional dysfunction	fibro-adipose substitution	none	frequent PVCs	negative family history, genetics n.a.
**ACM4**	male	43	dyskinesia, dilation and functional dysfunction	fibro-adipose substitution	inverted T waves in V1,V2,V3, V4 leads	arrhythmic storm, LBBB morphology VT	negative family history, no genetic mutations
**ACM5**	female	39	none	fibro-adipose substitution	inverted T waves in V1,V2 leads, low voltages	sporadic PVCs	affected relative, pathogenetic variant *PKP2*:c.2013delC

Table S2. Epigenetic drug library. Scheme of experimental plate 1 and plate 2 including the list of the 157 epigenetic drugs. AM: adipogenic medium; FM: profibrotic medium; MM: maintenance medium.

**Plate1**	**1**	**2**	**3**	**4**	**5**	**6**	**7**	**8**	**9**	**10**	**11**	**12**
**A**	*AM/FM untreated*	SB939	PCI 34051	4-iodo-SAHA	Sulforaphane	Sirtinol	C646	Garcinol	Ellagic Acid	Scriptaid	Suberohydroxamic Acid	*MM untreated*
**B**	*AM/FM untreated*	Apicidin	Cl-Amidine (hydrochloride)	F-Amidine (trifluoroacetate salt)	Unused	I-BET762	UNC0638	UNC669	CAY10669	Zebularine	Delphinidin (chloride)	*MM untreated*
**C**	*AM/FM DMSO*	ITF 2357	UNC0631	UNC0646	2,4-Pyridinedicarboxylic Acid (hydrate)	PFI-1	5-Azacytidine	SGI-1027	Decitabine	I-BET151	(+)-JQ1	*MM DMSO*
**D**	*AM/FM DMSO*	Sodium 4-Phenylbutyrate	IOX1	Gemcitabine	Unused	Daminozide	GSK-J1 (sodium salt)	GSK-J4 (hydrochloride)	CI-994	CPTH2 (hydrochloride)	Butyrolactone 3	*MM DMSO*
**E**	*AM/FM untreated (neg)*	Valproic Acid (sodium salt)	Tenovin-1	Tenovin-6 (hydrochloride)	BIX01294 (hydrochloride hydrate)	Anacardic Acid	AGK2	CAY10603	Splitomicin	CBHA	Salermide	*Unused*
**F**	*AM/FM untreated (neg)*	Pimelic Diphenylamide 106	Panobinostat	MS-275	HNHA	RG108	2',3',5'-triacetyl-5-Azacytidine	UNC0224	Chidamide	3-Deazaneplanocin A	5-Nitroso-8-quinolinol	*Unused*
**G**	*AM/FM DMSO (neg)*	Pyroxamide	N-Oxalylglycine	AMI-1 (sodium salt)	EPZ005687	SGC0946	UNC1215	AK-7	JNJ-26481585 (hydrochloride)	GSK343	Bromosporine	*Unused*
**H**	*AM/FM DMSO (neg)*	GSK2801	Plumbagin	SIRT1/2 Inhibitor IV	I-CBP112 (hydrochloride)	SGC-CBP30	UNC0642	UNC1999	(R)-PFI-2 (hydrochloride)	LMK 235	HPOB	*Unused*

**Plate2**	**1**	**2**	**3**	**4**	**5**	**6**	**7**	**8**	**9**	**10**	**11**	**12**
**A**	*AM/FM untreated*	HDAC6 Inhibitor	2-hexyl-4-Pentynoic Acid	PFI-3	JIB-04	CAY10683	GSK126	CPI-203	SP-2509	6-Thioguanine	Tubastatin A	*MM untreated*
**B**	*AM/FM untreated*	OTX015	A-366	OICR-9429	EPZ004777 (formate)	EPZ6438	EPZ5676	MC 1568	PBIT	Octyl-α-hydroxyglutarate	α-Hydroxyglutaric Acid (sodium salt)	*MM untreated*
**C**	*AM/FM DMSO*	UNC0379	RVX-208	CUDC-101	LAQ824	GSK-LSD1 (hydrochloride)	BVT 948	RGFP966	BG45	UMB-32	EPZ015666	*MM DMSO*
**D**	*AM/FM DMSO*	BI-2536	BAZ2-ICR	OG-L002	ML-324	GSK484 (hydrochloride)	Resminostat (hydrochloride)	NI-57	PFI-4	Tasquinimod	MM-102	*MM DMSO*
**E**	*AM/FM untreated (neg)*	TC-E 5003	L002	BI-9564	RN-1 (hydrochloride)	Mocetinostat	A-196	MS049 (hydrochloride)	GSK591	MS023 (hydrochloride)	PRT4165	*Unused*

**F**	*AM/FM untreated (neg)*	Unused	EPZ020411	GSK2879552	Cyproheptadine (hydrochloride hydrate)	CPTH6 (hydrobromide)	CeMMEC1	PCI 24781	BML-278	CeMMEC13	4'-bromo-Resveratrol	*Unused*
**G**	*AM/FM DMSO (neg)*	AZD 5153	Todralazine (hydrochloride)	HDAC3 Inhibitor	NCH-51	UF010	RGFP109	C-7280948	EED226	CAY10722	PAOA	*Unused*
**H**	*AM/FM DMSO (neg)*	TMP-195	Bufexamac	Trichostatin A	CAY10398	RSC-133	BML-210	CAY10591	(±)-EX-527	SAHA	Tranylcypromine (hydrochloride)	*Unused*

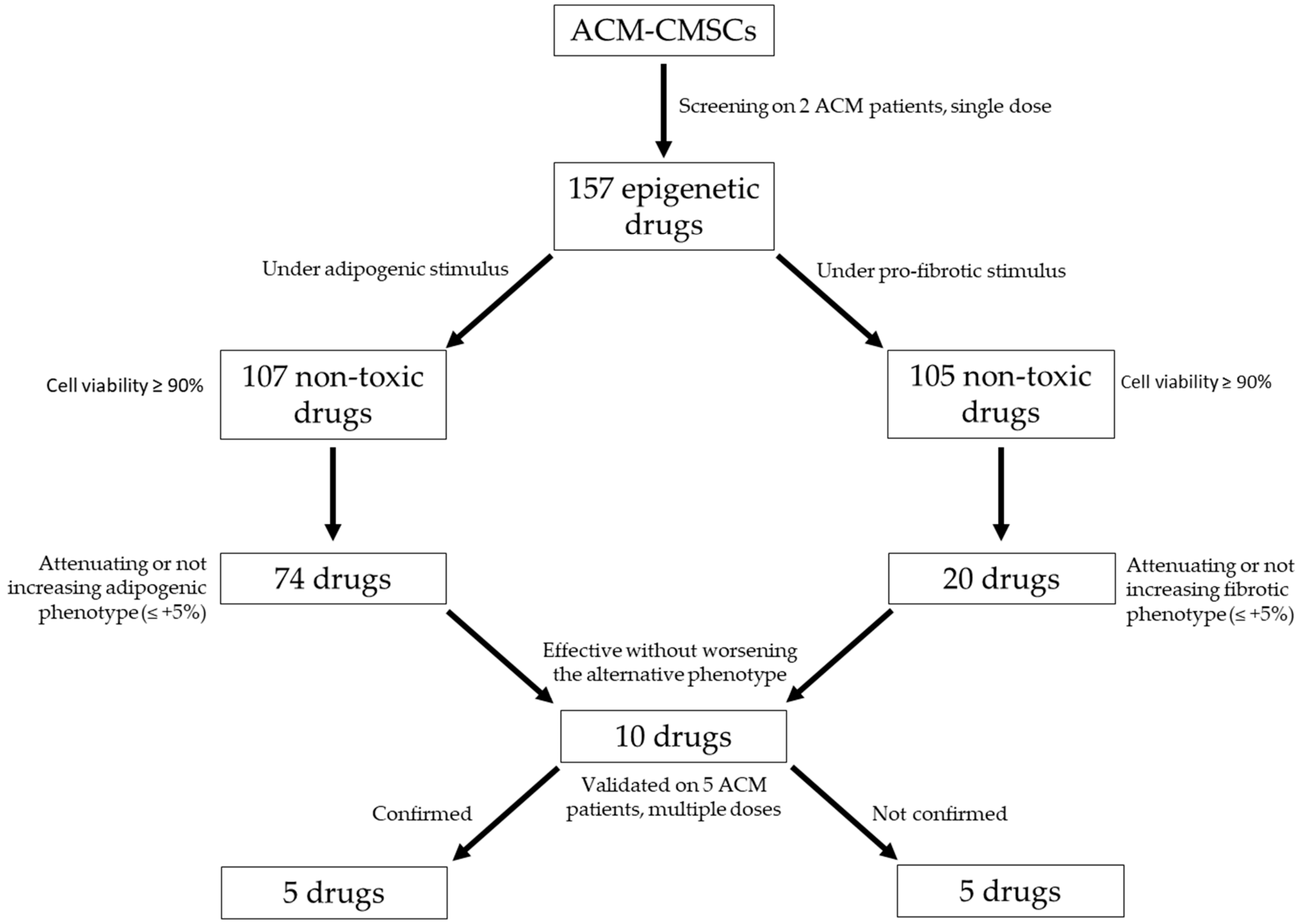

Figure S1. Drug screening flow chart. Flow chart of the selection of the epigenetic drugs. The screening has been performed on cardiac mesenchymal stromal cells (CMSCs) derived from 2 patients affected by arrhythmogenic cardiomyopathy (ACM), and validated on cells from 5 patients.

Table S3. Epigenetic drug screening. Table summarizing the effect of the epigenetic drugs on ACM CMSCs. Fold change (FC), respect to untreated, of Nile Red signal quantification, normalized on nuclei number, of CMSCs in adipogenic medium (AM) and in profibrotic medium (FM) treated with epidrugs is expressed as mean of the biological replicates (ACM1 and ACM2). FC, respect to untreated, of nuclei number of CMSCs in AM and in FM with epidrugs is expressed as mean of biological replicates. The mean of DMSO-treated controls was used as reference value. Values in green represent the achievement of the acceptable established thresholds: ≤ 5% for lipid and collagen signal; > -10% for nuclei number. Epigenetic drugs in yellow represent the selected drugs which reached the threshold criteria.

Epigenetic drugs	Mean of FC (%) NR/nuclei number (AM)	Mean of FC (%) nuclei number (AM)	Mean of FC (%) COL/nuclei number (FM)	Mean of FC (%) nuclei number (FM)
SB939	−3.81	−27.54	−1.00	−10.15
Apicidin	−48.45	−30.91	−12.89	−73.82
ITF 2357	−9.46	−20.31	11.64	−6.61
Sodium 4-Phenylbutyrate	59.60	0.50	18.35	6.78
Valproic Acid (sodium salt)	7.50	2.13	11.42	2.34
Pimelic Diphenylamide 106	7.64	−2.17	51.68	−13.25
Pyroxamide	−32.47	2.17	33.87	−13.65
GSK2801	−8.12	−9.23	70.68	−26.89
PCI 34051	39.79	3.42	38.66	6.42
Cl-Amidine (hydrochloride)	116.79	−3.82	19.01	11.80
UNC0631	74.22	−3.60	33.67	−3.44
IOX1	−57.58	−5.12	26.24	1.31
Tenovin-1	87.33	3.94	25.74	0.65
Panobinostat	−33.65	−82.44	163.17	−93.53
N-Oxalylglycine	3.14	−0.78	17.36	6.21
Plumbagin	−22.63	−2.16	82.25	−35.35
4-iodo-SAHA	−20.57	−9.61	33.96	−15.31
F-Amidine (trifluoroacetate salt)	11.49	−2.16	6.85	10.08
UNC0646	−6.01	3.46	19.39	8.03
Gemcitabine	−30.21	−17.81	26.34	−26.18
Tenovin-6 (hydrochloride)	−44.36	1.81	34.05	−3.65
MS-275	−57.80	−1.93	68.33	−22.42
AMI-1 (sodium salt)	12.05	6.54	21.35	−10.55
SIRT1/2 Inhibitor IV	−44.36	0.01	12.66	−8.49
Sulforaphane	−26.86	−0.75	15.13	−15.67
2,4-Pyridinedicarboxylic Acid (hydrate)	7.85	1.47	13.20	5.41
BIX01294 (hydrochloride hydrate)	11.27	−0.33	15.22	6.00
HNHA	64.21	5.28	32.32	−5.72
EPZ005687	34.48	−6.26	29.25	3.53
I-CBP112 (hydrochloride)	−5.07	−7.06	37.11	−27.65
Sirtinol	89.57	−4.89	26.41	−5.80
I-BET762	−12.96	−17.25	−16.17	−27.53
PFI-1	−14.88	−7.92	11.79	1.37
Daminozide	37.74	−1.24	20.33	−4.26
Anacardic Acid	8.03	−1.17	23.20	−4.75
RG108	10.49	−6.06	16.72	9.71
SGC0946	−6.18	−5.37	42.35	−6.05
SGC-CBP30	−27.90	−6.16	21.47	−3.45
C646	17.75	−1.53	14.61	−3.55
UNC0638	71.41	−5.92	28.48	4.76
5-Azacytidine	−24.62	−0.51	11.65	−16.67
GSK-J1 (sodium salt)	32.73	−4.95	7.99	8.98
AGK2	17.10	0.02	−12.55	−7.73

2',3',5'-triacetyl-5-Azacytidine	−26.34	3.85	16.18	−0.15
UNC1215	66.54	−6.10	24.82	2.01
UNC0642	−16.89	−20.48	64.22	−31.15
Garcinol	−5.81	−6.08	10.61	0.50
UNC669	84.24	2.32	18.56	17.04
SGI-1027	7.35	0.52	−11.06	16.24
GSK-J4 (hydrochloride)	4.17	−1.82	14.29	5.70
CAY10603	−26.99	−8.31	22.70	−3.80
UNC0224	32.63	−6.12	54.71	−23.05
AK-7	35.56	−7.35	64.57	−17.57
UNC1999	16.27	−10.72	55.94	−19.11
Ellagic Acid	17.65	−7.26	9.48	−2.62
CAY10669	20.16	−1.69	13.45	−3.95
Decitabine	−7.06	−5.70	16.45	10.08
CI-994	−20.24	−8.82	17.38	8.01
Splitomicin	−4.00	−1.59	5.00	13.18
Chidamide	−16.71	−9.43	42.07	−6.29
JNJ-26481585 (hydrochloride)	−33.19	−81.57	116.31	−90.49
(R)-PFI-2 (hydrochloride)	−12.16	−15.13	57.35	−35.26
Scriptaid	36.92	−7.79	−6.90	3.09
Zebularine	35.53	−10.61	27.25	−2.85
I-BET151	2.14	−15.35	−48.60	−23.88
CPTH2 (hydrochloride)	−27.52	0.36	−25.18	14.11
CBHA	14.15	−0.98	14.76	−8.27
3-Deazaneplanocin A	−0.76	−19.32	−13.22	−18.88
GSK343	1.96	−13.00	73.79	−23.08
LMK 235	40.57	−27.95	23.55	−29.78
Suberohydroxamic Acid	−14.65	−8.04	0.78	5.52
Delphinidin (chloride)	1.17	−2.46	38.62	−0.09
(+)-JQ1	−49.83	−60.21	−84.73	−52.47
Butyrolactone 3	1.09	−8.05	20.39	−7.17
Salermide	13.27	−9.30	25.17	9.24
5-Nitroso-8-quinolinol	90.46	−40.00	74.49	−23.79
Bromosporine	4.33	−32.98	−37.13	−15.26
HPOB	120.48	−35.34	−12.23	−50.46
HDAC6 Inhibitor	−21.16	−6.41	18.34	13.19
OTX015	−70.54	−64.83	−85.27	−37.90
UNC0379	6.01	3.93	19.48	3.01
BI-2536	−73.68	−30.36	−70.93	−17.42
TC-E 5003	−40.62	−9.74	12.90	−6.10
AZD 5153	−65.34	−72.20	−87.67	−50.82
TMP-195	−38.70	−8.72	26.66	−3.40
2-hexyl-4-Pentynoic Acid	−38.69	−6.78	13.08	12.32
A-366	−18.58	−1.15	21.86	13.77
RVX-208	−15.87	1.25	16.52	6.28
BAZ2-ICR	−52.66	4.00	11.71	7.83
L002	−52.68	4.05	30.73	5.26
EPZ020411	−4.76	−3.55	40.24	−11.19
Todralazine (hydrochloride)	−31.22	−3.09	24.57	1.05
Bufexamac	−33.88	−16.76	27.51	−17.89
PFI-3	−45.22	−12.04	18.89	14.69
OICR-9429	−2.14	1.57	33.05	3.24
CUDC-101	−55.04	−61.96	−33.48	−72.17
OG-L002	−20.86	4.16	31.95	2.53
BI-9564	−54.32	0.98	8.22	13.03
GSK2879552	−54.06	1.37	28.79	−11.26

HDAC3 Inhibitor	7.35	0.56	38.55	−1.82
Trichostatin A	−56.81	−23.35	−44.05	−37.55
JIB-04	−66.63	−69.50	−81.67	−6.27
EPZ004777 (formate)	−18.29	−8.47	18.38	16.12
LAQ824	−94.37	−99.38	−59.77	−73.40
ML-324	−5.38	5.15	33.59	13.43
RN-1 (hydrochloride)	−56.79	−4.82	11.56	9.56
Cyproheptadine (hydrochloride hydrate)	−19.85	−1.22	46.26	−1.97
NCH-51	−56.57	7.04	44.29	−7.11
CAY10398	−51.13	−2.53	−13.34	−22.54
CAY10683	−19.03	−6.86	8.00	12.62
EPZ6438	−17.24	−3.67	18.59	10.38
GSK-LSD1 (hydrochloride)	−51.98	−7.47	12.99	10.40
GSK484 (hydrochloride)	−31.05	4.44	9.39	14.98
Mocetinostat	−45.04	−10.30	0.36	−5.18
CPTH6 (hydrobromide)	−50.56	12.87	3.53	19.76
UF010	−27.07	−28.55	6.84	−3.36
RSC-133	−39.06	−23.95	9.95	−15.27
GSK126	−38.94	−5.59	1.02	12.37
EPZ5676	−49.14	3.94	5.00	17.27
BVT 948	−81.41	8.69	−8.70	19.32
Resminostat (hydrochloride)	−55.07	−6.42	9.79	6.26
A-196	−53.92	3.80	9.99	7.55
CeMMEC1	−28.99	−3.36	23.77	8.23
RGFP109	−30.43	11.76	32.73	−14.90
BML-210	−38.86	−9.89	14.08	−15.68
CPI-203	−64.64	−61.81	−82.71	−28.01
MC 1568	−51.48	−8.30	15.94	6.58
RGFP966	−48.21	2.94	23.80	7.54
NI-57	−49.06	−2.47	−0.02	21.58
MS049 (hydrochloride)	−35.51	−1.24	14.68	3.53
PCI 24781	−49.33	−27.72	−18.39	−10.08
C-7280948	−18.57	−31.85	13.00	−15.20
CAY10591	−19.56	−21.93	31.74	−20.10
SP-2509	−63.06	−54.35	3.31	−8.80
PBIT	−29.46	−9.30	3.11	14.21
BG45	−36.03	−7.80	13.92	5.22
PFI-4	−15.69	−20.98	6.47	14.86
GSK591	−1.22	2.47	11.46	−8.33
BML-278	−22.15	−3.48	12.87	9.15
EED226	−29.34	5.19	16.58	−20.16
(±)-EX-527	−42.99	−8.97	4.01	−31.15
6-Thioguanine	−61.09	−41.18	2.17	15.85
Octyl-α-hydroxyglutarate	−51.92	−20.95	5.42	18.62
UMB-32	−27.53	−30.63	−16.78	17.65
Tasquinimod	−54.29	−10.20	7.55	16.08
MS023 (hydrochloride)	−41.15	−7.87	5.77	5.43
CeMMEC13	−25.95	5.29	22.88	−10.94
CAY10722	−45.88	1.55	−11.63	−4.14
SAHA	−29.57	−13.95	−11.72	−23.44
Tubastatin A	−50.86	−62.28	2.56	14.89
α-Hydroxyglutaric Acid (sodium salt)	−38.26	−53.85	9.69	11.30
EPZ015666	−37.74	−54.35	12.25	3.58
MM-102	−11.37	−47.31	4.24	3.06
PRT4165	−42.09	−43.52	20.59	−4.98
4'-bromo-Resveratrol	−32.79	−46.76	−3.16	−17.34

PAOA	−30.65	−49.47	23.02	−9.97
Tranylcypromine (hydrochloride)	−44.89	−61.49	18.22	−25.59

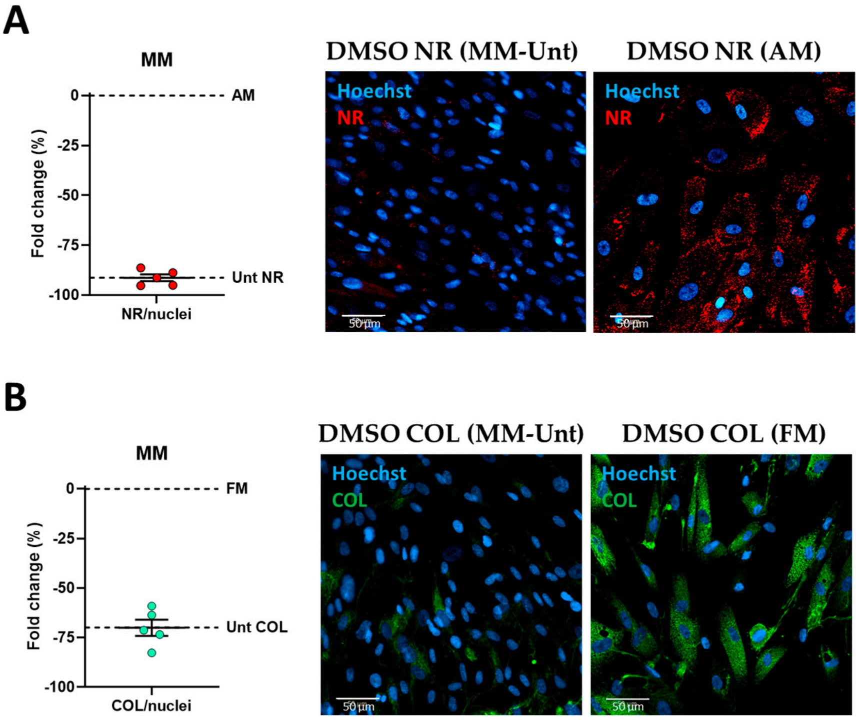

Figure S2. Nile red and collagen signal of DMSO-treated CMSCs in maintenance medium and under adipogenic or profibrotic stimuli. (A) Graphical representation of the fold change percentage of Nile Red (NR) signal quantification normalized on nuclei number, for ACM CMSCs treated with DMSO in maintenance medium (MM; untreated, Unt), respect to the reference value in adipogenic medium (AM), with the relative representative fluorescent images (NR in red, Hoechst in blue). (B) Graphical representation of the fold change percentage of collagen (COL) signal quantification normalized on nuclei number, for ACM CMSCs treated with DMSO in MM, respect to the reference value in profibrotic medium (FM), with the relative representative fluorescent images (COL in green, Hoechst in blue). The mean of DMSO-treated controls in AM and FM was used as reference and set to zero, and it is represented by a dotted line. In the graphs, biological replicates with the relative means ± standard errors are shown. The mean values of the DMSO-treated cells in MM have been used as untreated control in the graphs of Figure 2 and Figure S4.

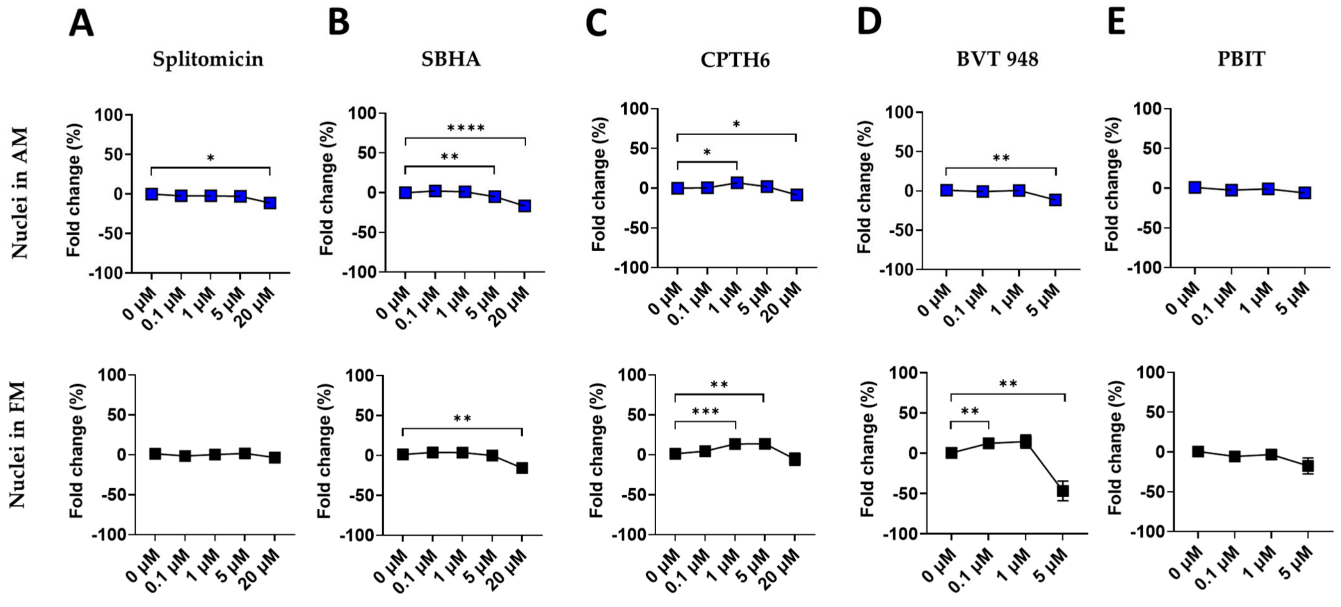

Figure S3. Effect of splitomicin, SBHA, CPTH6, BVT-948 and PBIT on nuclei count of ACM CMSCs. Graphical representation of the effect of the effective epigenetic drugs on ACM CMSCs from 5 patients (ACM1-5). (A) Fold change of nuclei count in adipogenic medium (AM, in blue) and in profibrotic medium (FM, in black) for CMSCs treated with splitomicin; (B) suberohydroxic acid (SBHA); (C) CPTH6; (D) BVT-948; (E) PBIT. The mean of DMSO-treated controls in AM and FM was used as reference and set to zero. In the graphs, each dot represents the mean of biological replicates ± standard errors (n=5). Paired two-tailed t-test was used for comparisons: * = p value <0.05; ** = p value < 0.005; *** = p value < 0.0005; **** = p value < 0.0001.

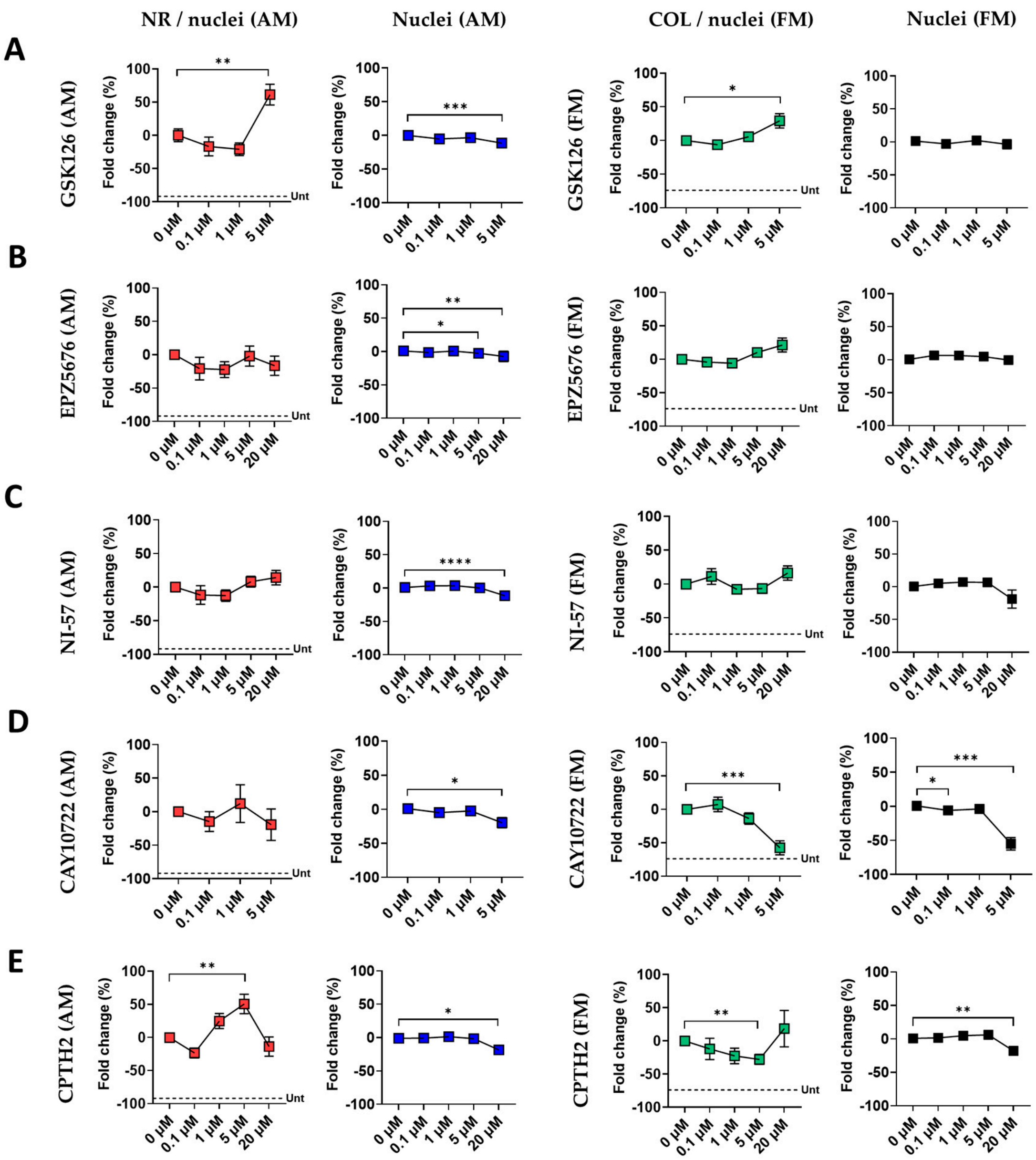

Figure S4. Effect of GSK126, EPZ5676, NI-57, CAY10722 and CPTH2 on ACM CMSCs. Graphical representation of the effect of the non-validated epigenetic drugs on ACM CMSCs. (A) Fold change percentage of Nile Red (red) or collagen (green) signal quantification normalized on nuclei number, and fold change of nuclei count in AM (blue) and in FM (black) for CMSCs treated with GSK126; (B) EPZ5676; (C) NI-57; (D) CAY10722; (E) CPTH2. The mean of the DMSO-treated controls in MM is represented by a dotted line (Unt, untreated in C to G). In the graphs, each dot represents the mean of biological replicates ± standard errors (n=5). Paired two-tailed t-test was used for comparisons: * = p value <0.05; ** = p value < 0.005; *** = p value < 0.0005; **** = p value < 0.0001.

Table S4. Effect of GSK126, EPZ5676, NI-57, CAY10722 and CPTH2 on ACM CMSCs. Table summarizing the effect of the non-validated epigenetic drugs on ACM CMSCs from 5 patients (ACM1-5). Fold change (FC) percentage of Nile Red signal quantification normalized on nuclei number of CMSCs in adipogenic medium (AM) and of collagen (COL) in profibrotic medium (FM) treated with epidrugs is expressed as mean of biological replicates ± standard error; the mean of the controls in AM and FM was used as reference value. Fold change percentage of nuclei number of CMSCs in AM and in FM with selected epidrugs is expressed as mean of biological replicates; the mean of DMSO-treated controls in AM and FM was used as reference value (0%).

EPIGENETIC DRUG	Dose	Fold change % Nile red / nuclei number (AM)		Fold change % nuclei number (AM)		Fold change % collagen / nuclei number (FM)		Fold change % nuclei number (FM)	
		Mean	SEM	Mean	SEM	Mean	SEM	Mean	SEM
**GSK126**	0.1 μM	−16.7	14.1	−5.2	3.1	−6.3	6.2	−3.0	4.8
	1 μM	−20.9	9.4	−3.5	3.3	5.5	5.4	2.3	3.5
	5 μM	61.4	15.7	−11.5	2.3	29.2	10.5	−3.6	7.0
**EPZ5676**	0.1 μM	−20.8	16.9	−1.4	1.8	−4.3	6.7	6.5	2.8
	1 μM	−22.3	11.8	0.8	1.6	−6.0	5.6	6.4	2.7
	5 μM	−2.1	14.9	−2.6	1.2	10.0	5.6	4.7	2.3
	20 μM	−16.5	14.5	−7.3	2.5	21.3	10.4	−0.4	3.2
**NI-57**	0.1 μM	−11.7	13.8	3.3	2.5	11.2	11.6	5.3	2.2
	1 μM	−12.6	7.5	3.6	2.0	−7.7	4.2	7.3	2.3
	5 μM	8.1	7.9	0.3	1.1	−6.6	3.5	6.8	3.5
	20 μM	14.2	10.8	−11.4	1.2	16.3	9.4	−18.8	13.8
**CAY10722**	0.1 μM	−14.8	14.1	−5.0	3.1	7.2	10.8	−6.1	2.0
	1 μM	12.1	28.1	−2.5	2.3	−13.8	8.6	−4.0	2.6
	5 μM	−19.4	23.5	−19.8	7.5	−57.6	10.5	−55.1	9.1
**CPTH2**	0.1 μM	−23.4	7.5	−0.7	4.1	−12.1	16.0	1.6	3.1
	1 μM	24.7	10.6	1.4	3.4	−22.6	11.8	4.7	5.5
	5 μM	50.6	13.7	−1.6	3.3	−27.8	7.5	6.1	3.6
	20 μM	−13.8	12.6	−18.5	6.2	18.5	27.4	−17.8	5.2
